# Supplementary figures and images for: A pyridinesulfonamide derivative FD268 suppresses cell proliferation and induces apoptosis via inhibiting PI3K pathway in acute myeloid leukemia
Source: PLoS One. 2022 Nov 22;17(11):e0277893. doi: 10.1371/journal.pone.0277893 (PMC9681083; doi:10.1371/journal.pone.0277893)

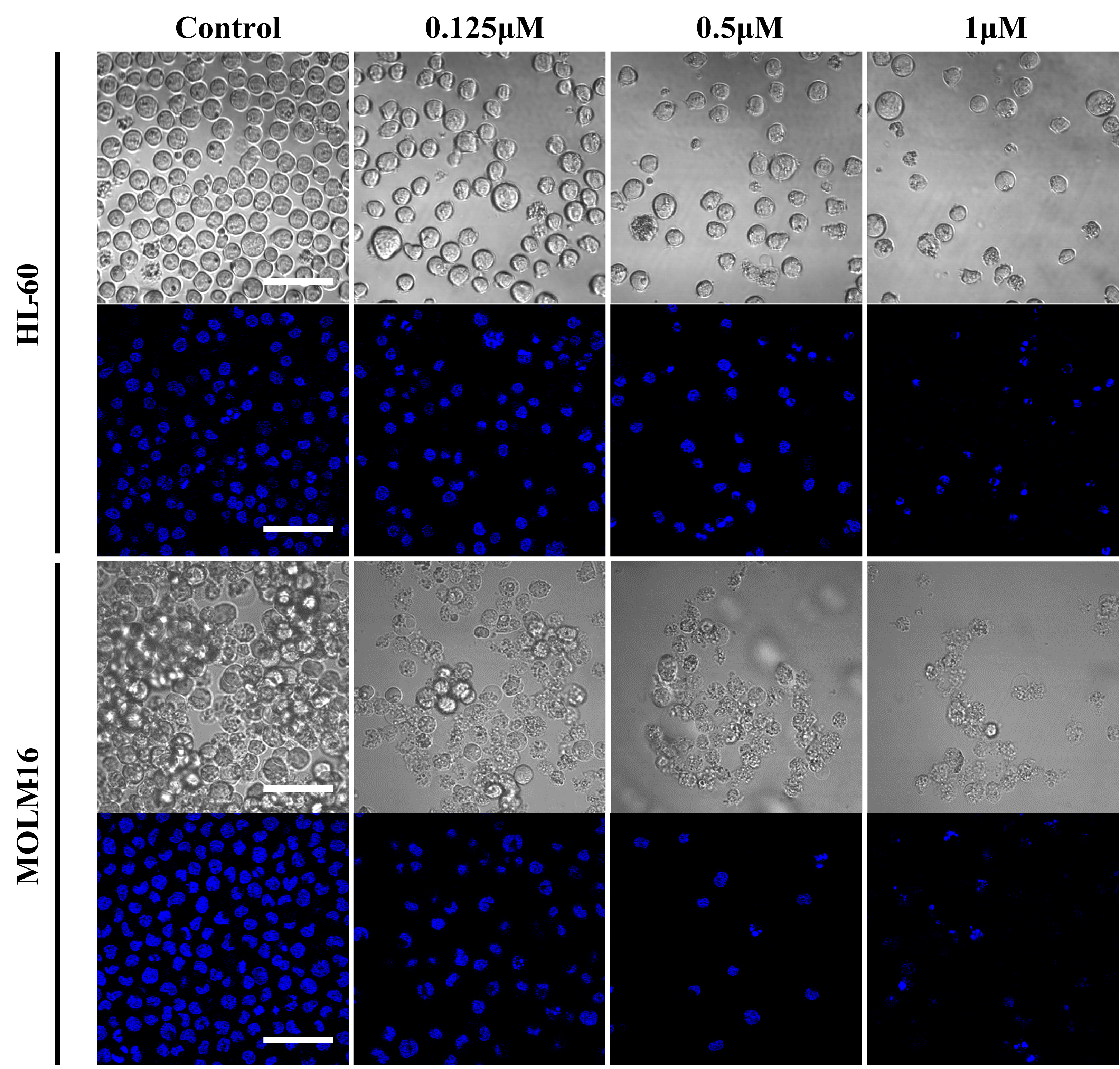

Supplement: S1 Fig — Cellular morphology of HL-60 and MOLM-16 treated with various concentrations of FD268 at 48 h. Phase contrast microscopy images and fluorescence microscopy images after staining with DAPI were observed. Cells incubated with DMSO (0.01%) were used control. Scale bar: 20 μm. (TIF) [file pone.0277893.s001.tif]

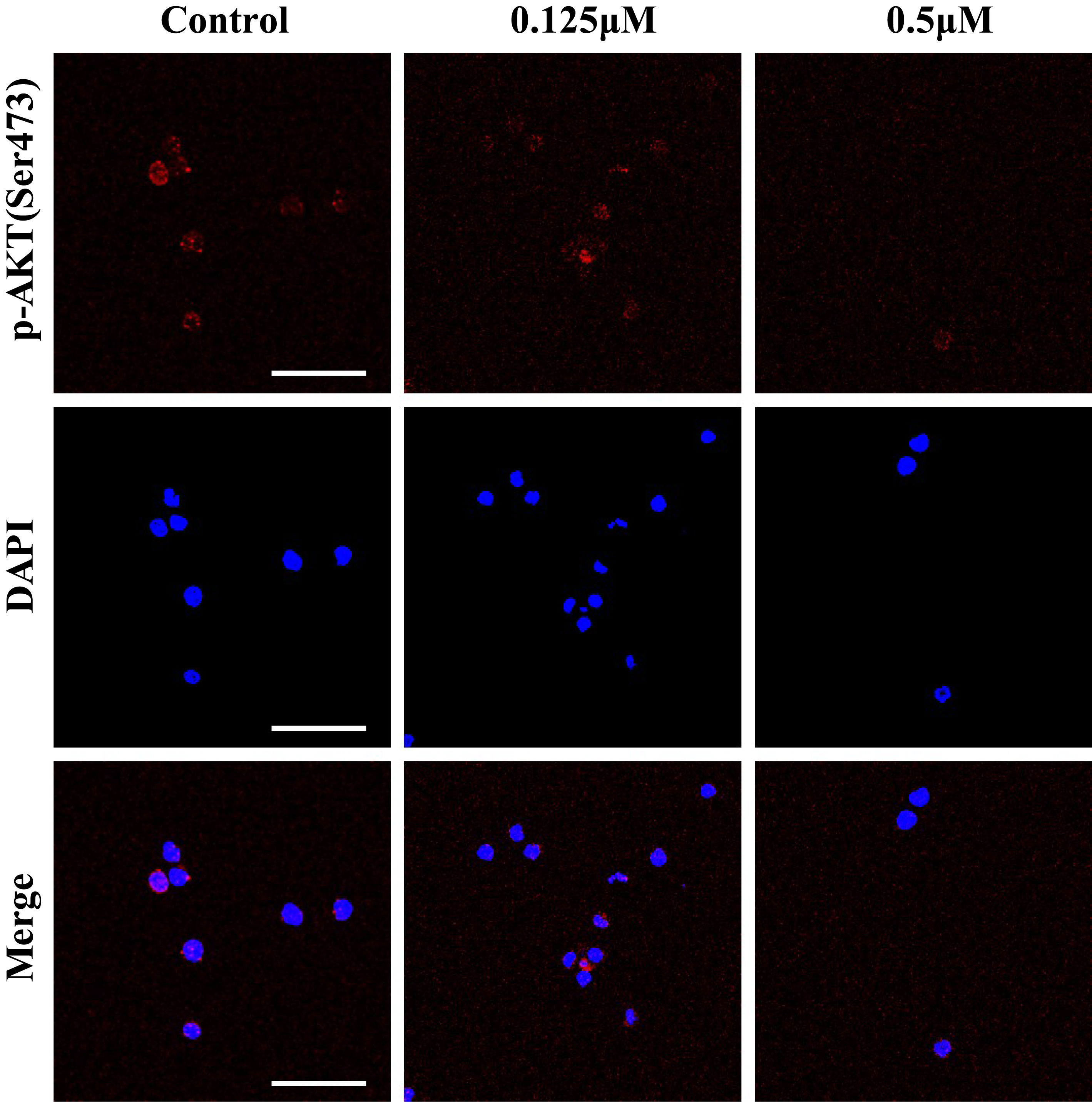

Supplement: S2 Fig — HL-60 cells were treated with indicated concentrations of FD268 for 24 h. The p-AKT(Ser473) expression and location was detected by immunofluorescence assay. The fluorescence microscopy images after staining with DAPI were observed. Scale bar: 20 μm. (TIF) [file pone.0277893.s002.tif]

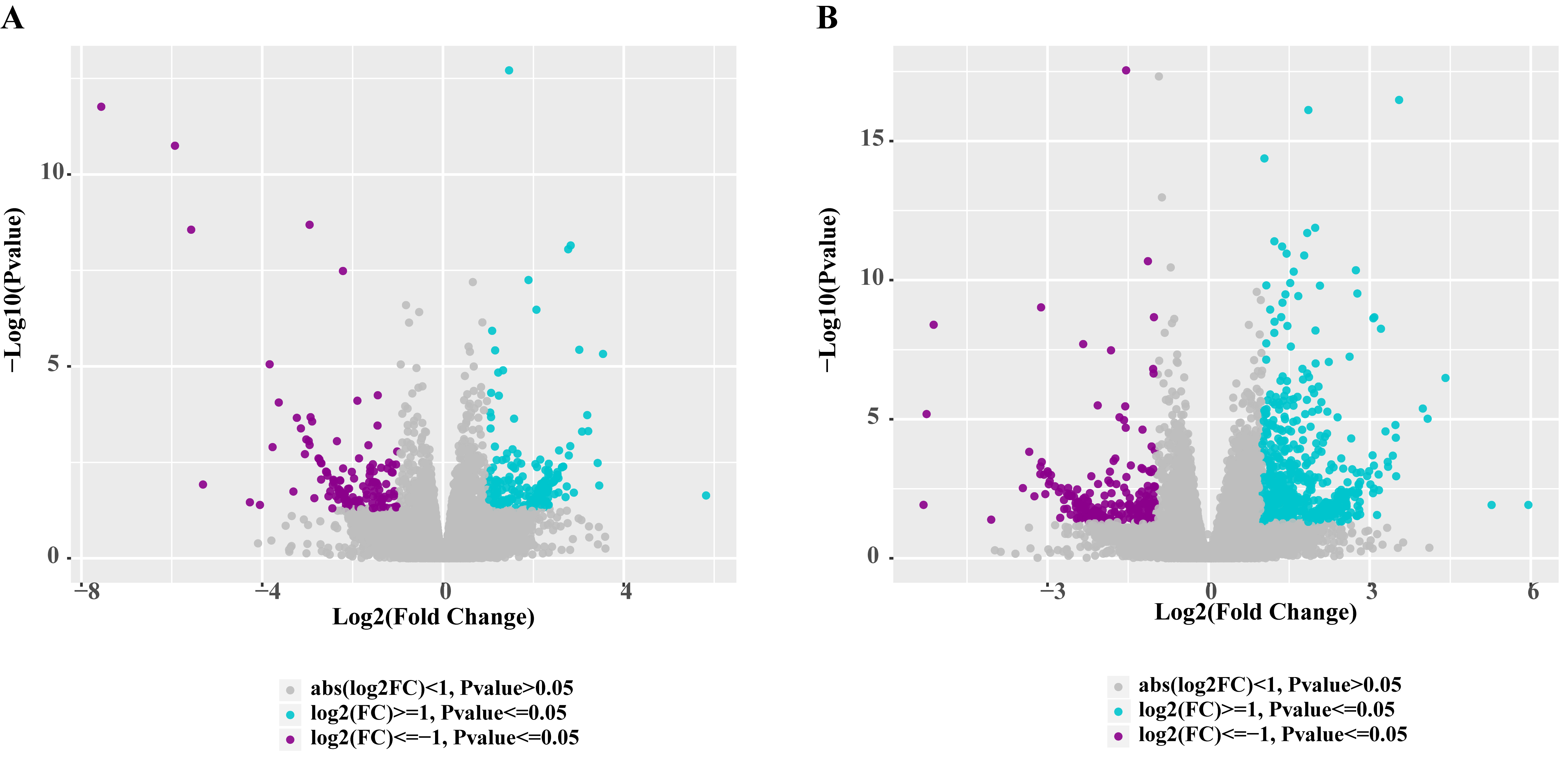

Supplement: S3 Fig — DESeq2 was used to screen differentially expressed genes among different groups (|log2FC| ≥1and P value≥0.05). Volcano plot of deferentially expressed genes. (A) 0.125 μM FD268-treated group. (b) 0.5 μM FD268-treated group. 194 up-regulated and 184 down-regulated mRNAs were screened by comparison in group FD268-0.125μM, 655 up-regulated and 240 down-regulated mRNAs were screened by comparison in group FD268-0.5μM. (TIF) [file pone.0277893.s003.tif]

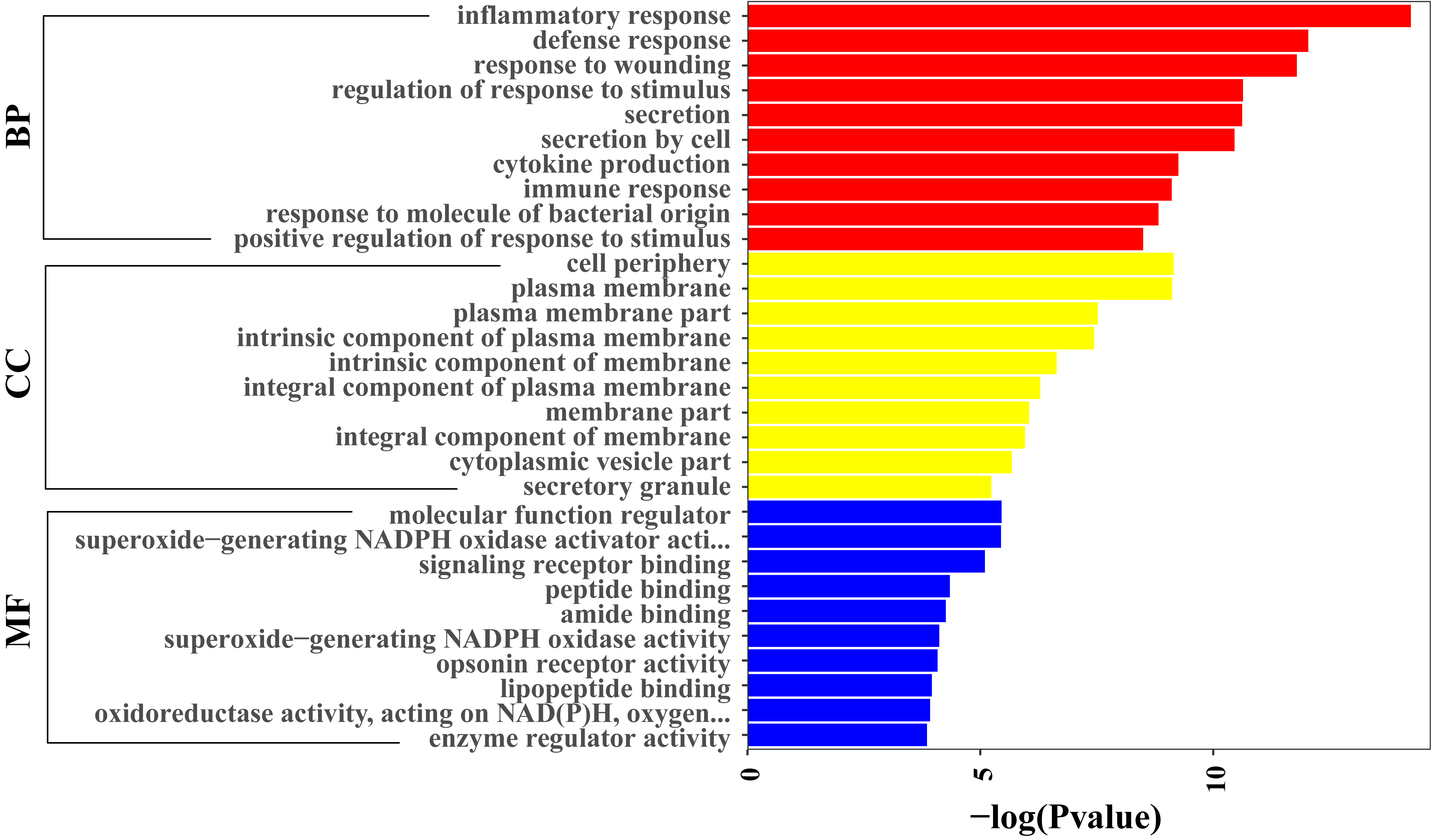

Supplement: S4 Fig — The top ten significantly enriched biological processes (BP), cell component (CC), molecule function (MF) are shown. (TIF) [file pone.0277893.s004.tif]

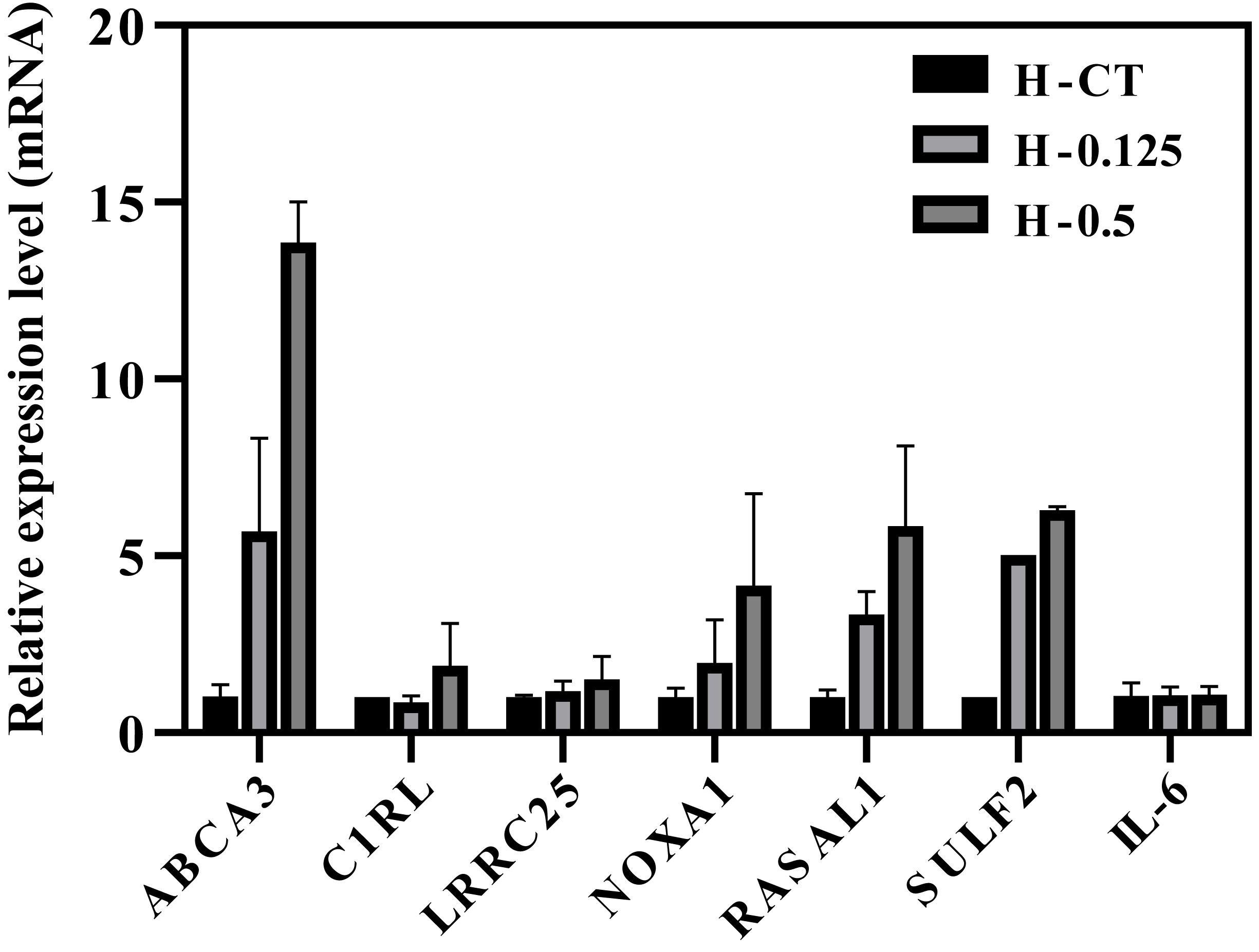

Supplement: S5 Fig — HL-60 cells were treated by FD268 (0.125μM, 0.5 μM) for 24 h. The expression of target genes ABCA3, C1RL, LRRC25, NOXA1, RASAL1, AULF2 and IL-6 in HL-60 cells were determined using qRT-PCR analysis. (TIF) [file pone.0277893.s005.tif]
