# Supplementary material for: A pyridinesulfonamide derivative FD268 suppresses cell proliferation and induces apoptosis via inhibiting PI3K pathway in acute myeloid leukemia
Source: PLoS One. 2022 Nov 22;17(11):e0277893. doi: 10.1371/journal.pone.0277893 (PMC9681083; doi:10.1371/journal.pone.0277893)
Supplement: S1 File — (DOCX) [file pone.0277893.s010.docx]

**Supporting information**


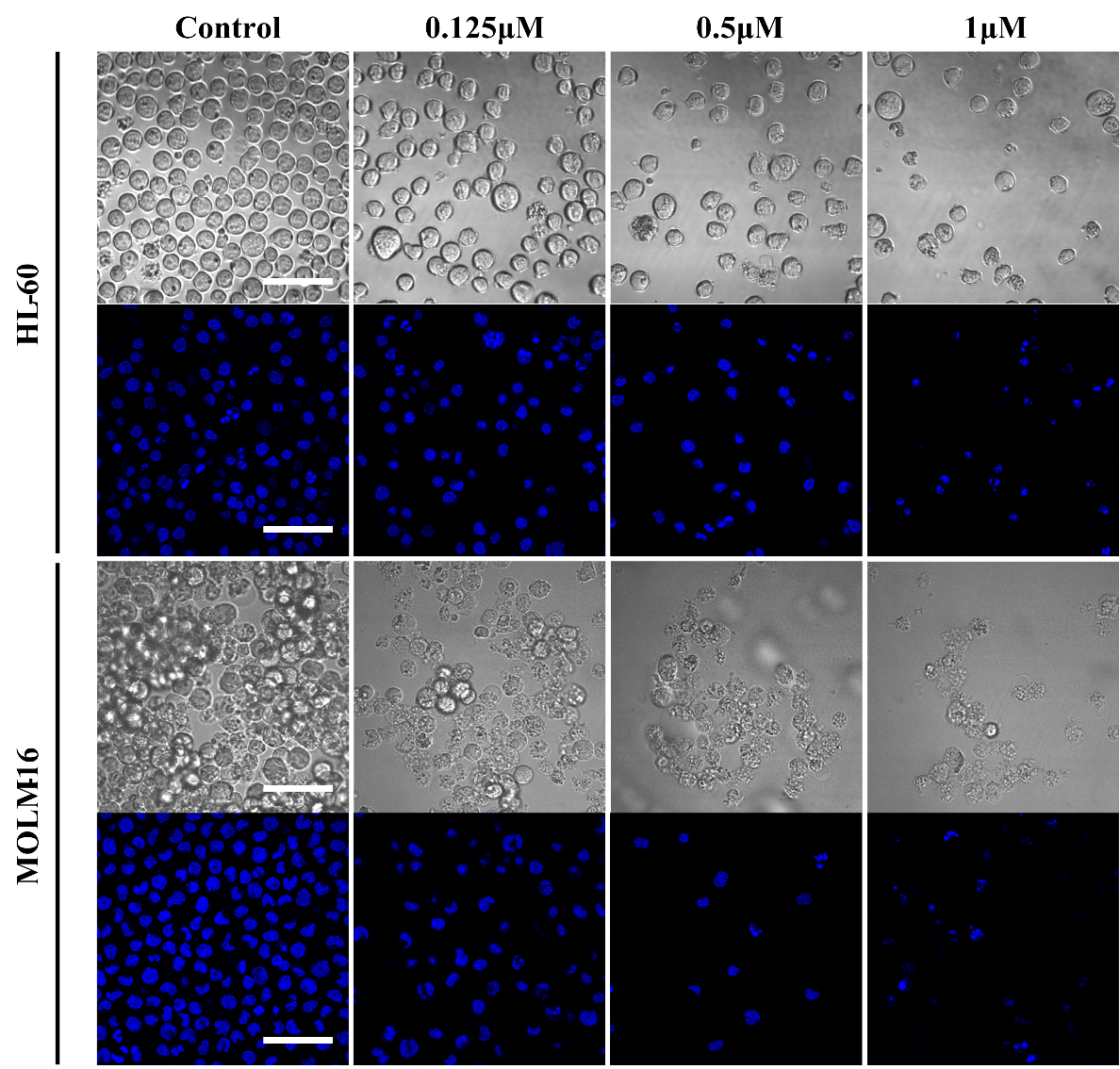


**S1 Fig. morphological changes HL-60 and MOLM-16 treated with FD268 for 48h.** Cellular morphology of HL-60 and MOLM-16 treated with various concentrations of FD268 at 48 h. Phase contrast microscopy images and fluorescence microscopy images after staining with DAPI were observed. Cells incubated with DMSO (0.01%) were used control. Scale bar: 20 μm.


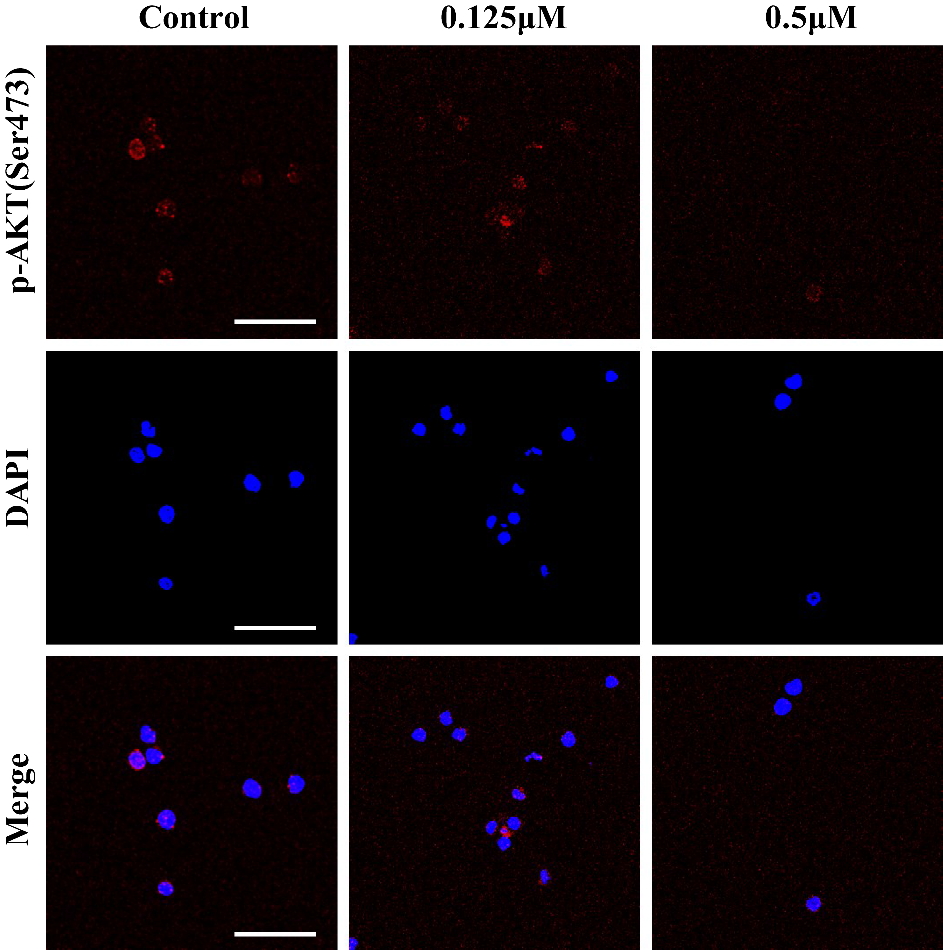


**S2 Fig. FD268 concentration-dependently inhibited the phosphorylation of AKT (Ser473) in HL-60 cells.** HL-60 cells were treated with indicated concentrations of FD268 for 24 h. The p-AKT(Ser473) expression and location was detected by immunofluorescence assay. The fluorescence microscopy images after staining with DAPI were observed. Scale bar: 20 μm.


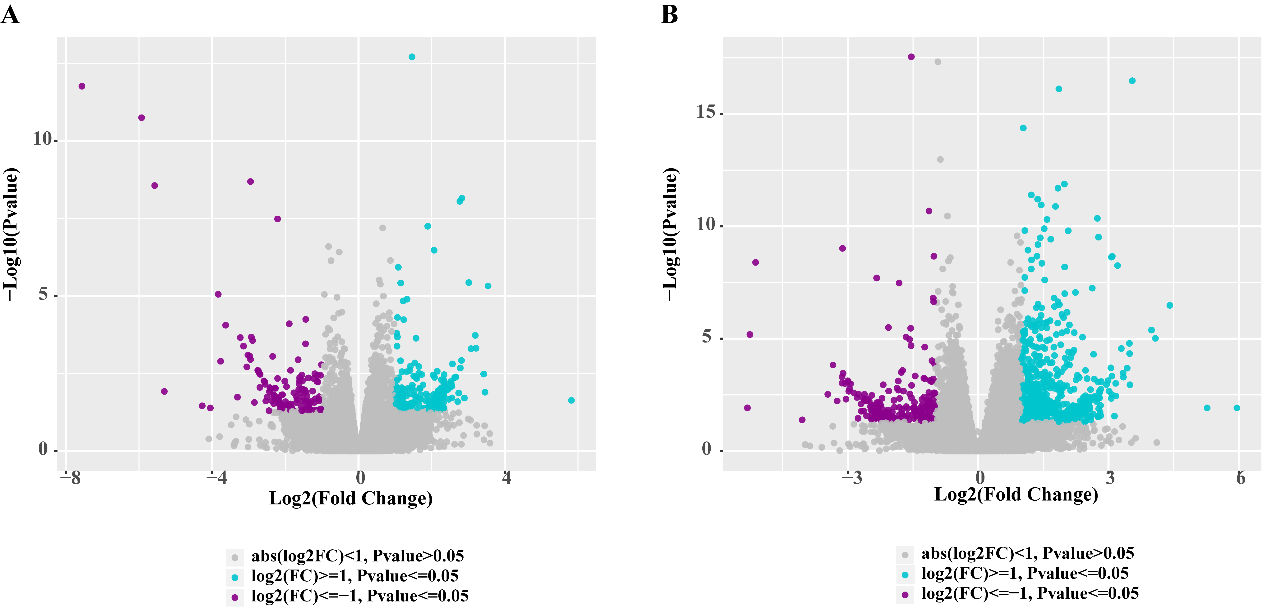
 **S3 Fig. Differentially expressed gene (DEGs) in HL-60 cells treated with FD268.** DESeq2 was used to screen differentially expressed genes among different groups (|log2FC| ≥1and P value≥0.05). Volcano plot of deferentially expressed genes. (A) 0.125 μM FD268-treated group. (b) 0.5 μM FD268-treated group. 194 up-regulated and 184 down-regulated mRNAs were screened by comparison in group FD268-0.125μM，655 up-regulated and 240 down-regulated mRNAs were screened by comparison in group FD268-0.5μM.


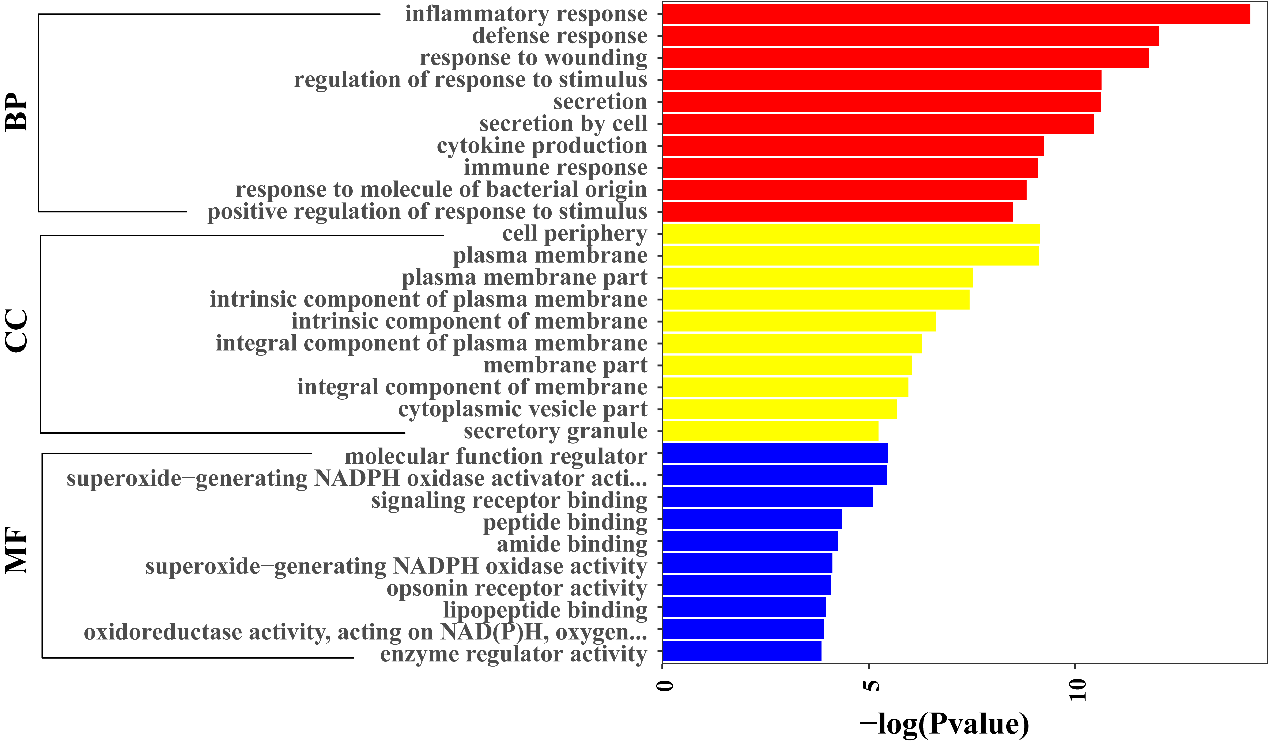


**S4 Fig. Gene Ontology (GO) analysis of RNA-seq data.** The top ten significantly enriched biological processes (BP), cell component (CC), molecule function (MF) are shown.


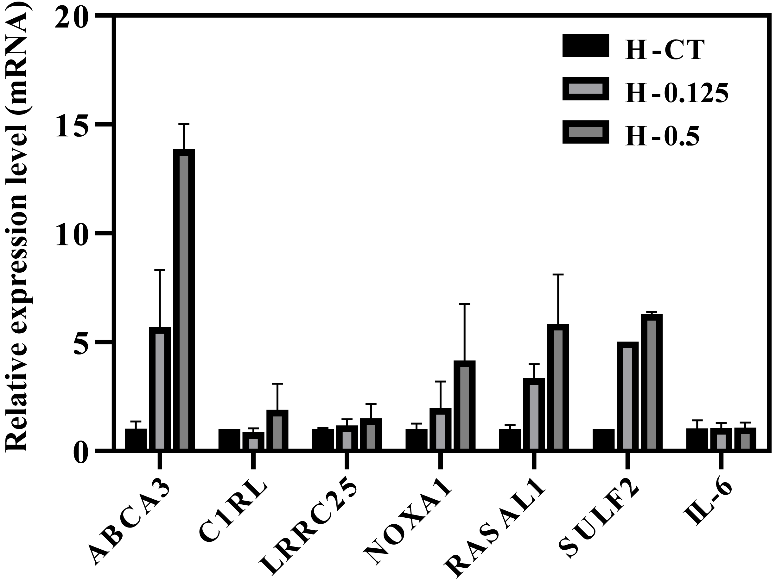


**S5 Fig. Expression level of overlapped common of target genes dose-dependently regulated by FD268.** HL-60 cells were treated by FD268 (0.125μM ,0.5 μM) for 24 h. The expression of target genes ABCA3, C1RL, LRRC25, NOXA1, RASAL1, AULF2 and IL-6 in HL-60 cells were determined using qRT-PCR analysis.

**S1 Table. The sequence of primers used for q-PCR.**

| Gene | Forward primer (5′-3′) | Reverse primer (5′-3′) | Size  (bp) |
| --- | --- | --- | --- |
| HPRT | TGGACAGGACTGAACGTCTTG | CCAGCAGGTCAGCAAAGAATTTA | 111 |
| Bcl-2 | CGGTGGGGTCATGTGTGTG | CGGTTCAGGTACTCAGTCATCC | 90 |
| MCL-1 | AGAAAGCTGCATCGAACCAT | CCAGCTCCTACTCCAGCAAC | 183 |
| Bad | CCCAGAGTTTGAGCCGAGTG | CCCATCCCTTCGTCGTCCT | 249 |
| CDK2 | TTGACGGGAGAAGTTGTGG | TGATGAGGGGAAGAGGAATG | 256 |
| p21cip1 | GGACATGTGCACGGAAGGA | GAAGCTTGGCAAAGGGCCTG | 221 |
| p27kip1 | TCAGACGGTTCCCCAAATGC | AGTGCGTGCTCCTTTAGTGAT | 112 |
| ABCA3 | CTCGCTGTTCCTCAAGCAGA | CTTCAAACCTGTGCGTGCTC | 185 |
| C1RL | CTGCCAAGGTCCAGAACCACTGC | TCAATTCTTGCCATTCATCACTCCCTTGATCC | 122 |
| LRRC25 | CCCTCCACTCCCGACTATGAG | TGTCCTCTGAAGGGTGAGCC | 91 |
| NOXA1 | CTTGCACCTCTTCTCGGGC | TGGTCACGGCTTGGTCAAAT | 122 |
| RASAL1 | TGGATTTCTCTTCTTGCGATTCT | TGTTGGTCCCGAAGGTCAA | 72 |
| SULF2 | GTGACACATCGGTGCTACATC | CCTTTGTGCTGGGTGTGGTA | 224 |
| IL-6 | TACAGGGAGAGGGAGCGATA | CAGGGAGAAGGCAACTGGAC | 207 |

**S2 Table. A total of terms annotated and categorized for the identified differential target genes.**

**S3 Table. KEGG pathways annotated and categorized for the identified differential target genes.**

**S4 Table. Target genes identified by overlapping the deferentially expressed of 0.125 µM FD268-treated and 0.5 µM FD268-treated group.**
