# Supplementary material for: A pyridinesulfonamide derivative FD268 suppresses cell proliferation and induces apoptosis via inhibiting PI3K pathway in acute myeloid leukemia
Source: PLoS One. 2022 Nov 22;17(11):e0277893. doi: 10.1371/journal.pone.0277893 (PMC9681083; doi:10.1371/journal.pone.0277893)
Supplement: S1 Raw images — (PDF) [file pone.0277893.s011.pdf]

# Original Images for Blots

**The images were scanned with FUSION FX software (Vilber Lourmat Inc., France)**

Figure. 3C

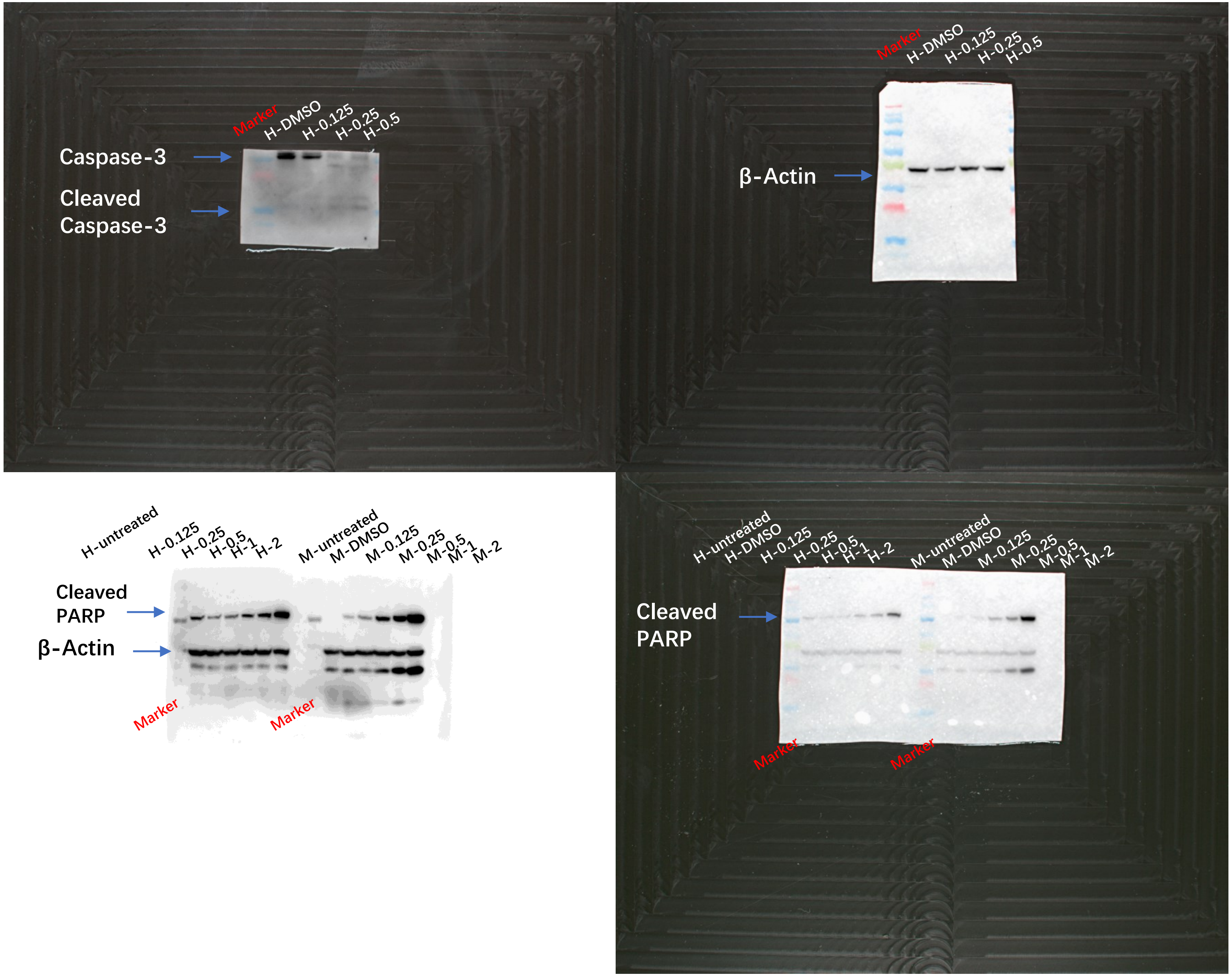

Marker
H-DMSO
H-0.125
H-0.25
H-0.5
Caspase-3
Cleaved Caspase-3
β-Actin
H-untreated
H-0.125
H-0.25
H-0.5
H-1
H-2
M-untreated
M-DMSO
M-0.125
M-0.25
M-0.5
M-1
M-2
Cleaved PARP
H-DMSO
Cleaved PARP

Figure. 3C

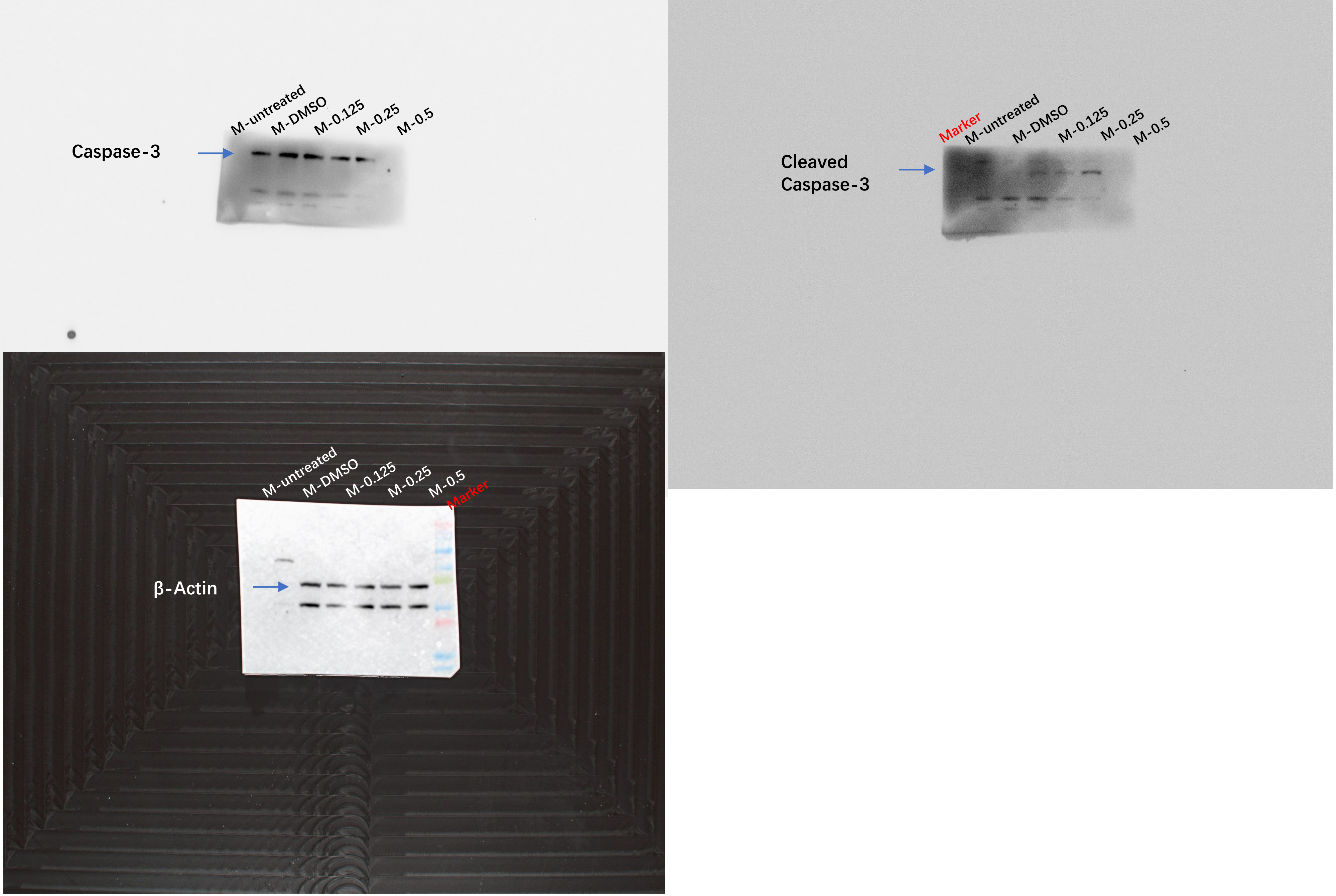
M-untreated
M-DMSO
M-0.125
M-0.25
M-0.5
Caspase-3
Marker
M-untreated
M-DMSO
M-0.125
M-0.25
M-0.5
Cleaved
Caspase-3
M-untreated
M-DMSO
M-0.125
M-0.25
M-0.5
Marker
β-Actin

## Figure. 4C

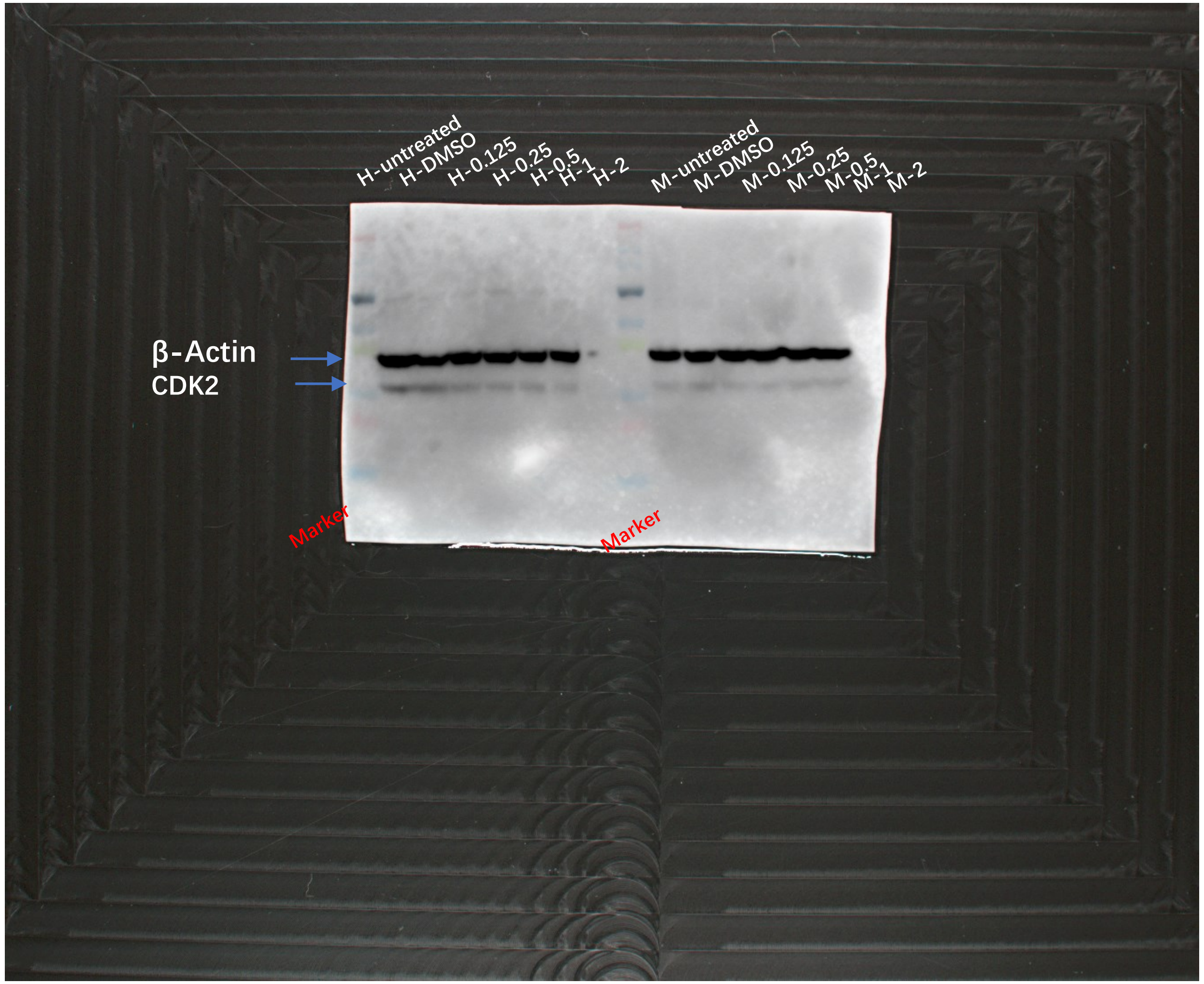
H-untreated
H-DMSO
H-0.125
H-0.25
H-0.5
H-1
H-2
M-untreated
M-DMSO
M-0.125
M-0.25
M-0.5
M-1
M-2
β-Actin
CDK2
Marker
Marker

Figure. 5C

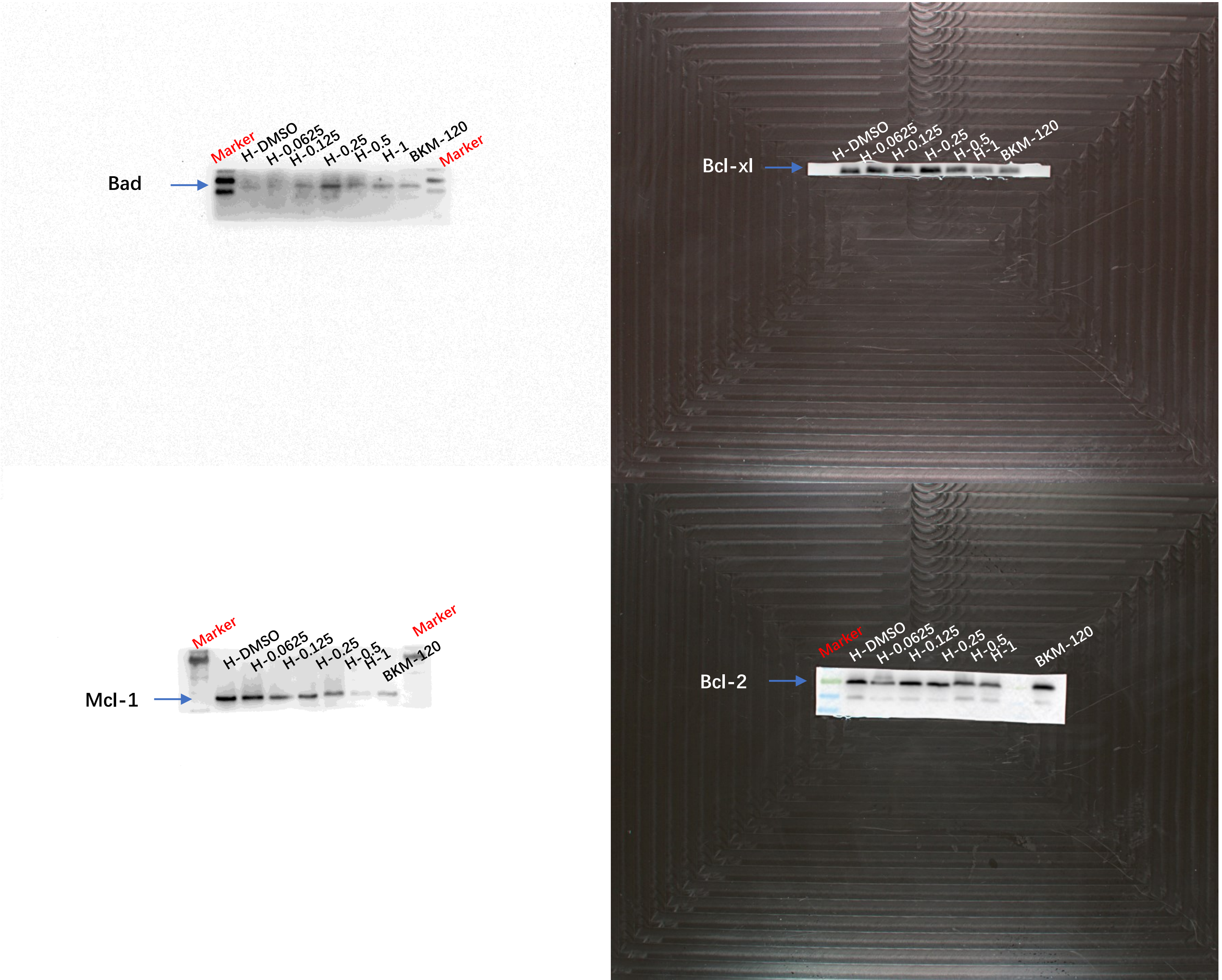
Bad
Marker
H-DMSO
H-0.0625
H-0.125
H-0.25
H-0.5
H-1
BKM-120
Marker
Bcl-xl
H-DMSO
H-0.0625
H-0.125
H-0.25
H-0.5
H-1
BKM-120
Mcl-1
Marker
H-DMSO
H-0.0625
H-0.125
H-0.25
H-0.5
H-1
BKM-120
Marker
Bcl-2
Marker
H-DMSO
H-0.0625
H-0.125
H-0.25
H-0.5
H-1
BKM-120

# Figure. 5C

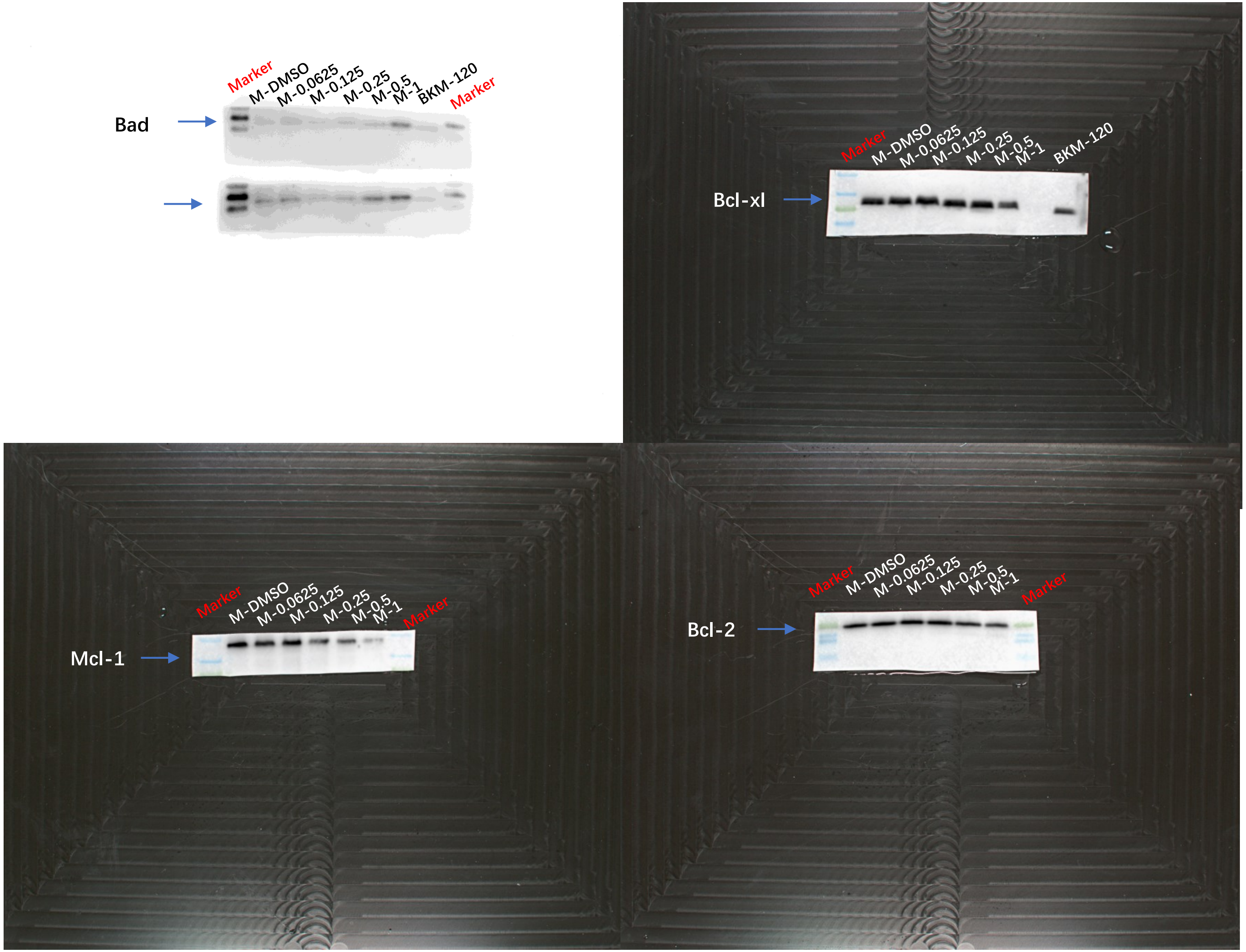
Marker
M-DMSO
M-0.0625
M-0.125
M-0.25
M-0.5
M-1
BKM-120
Marker
Bad
Bcl-xl
Mcl-1
Bcl-2

# Figure. 5C

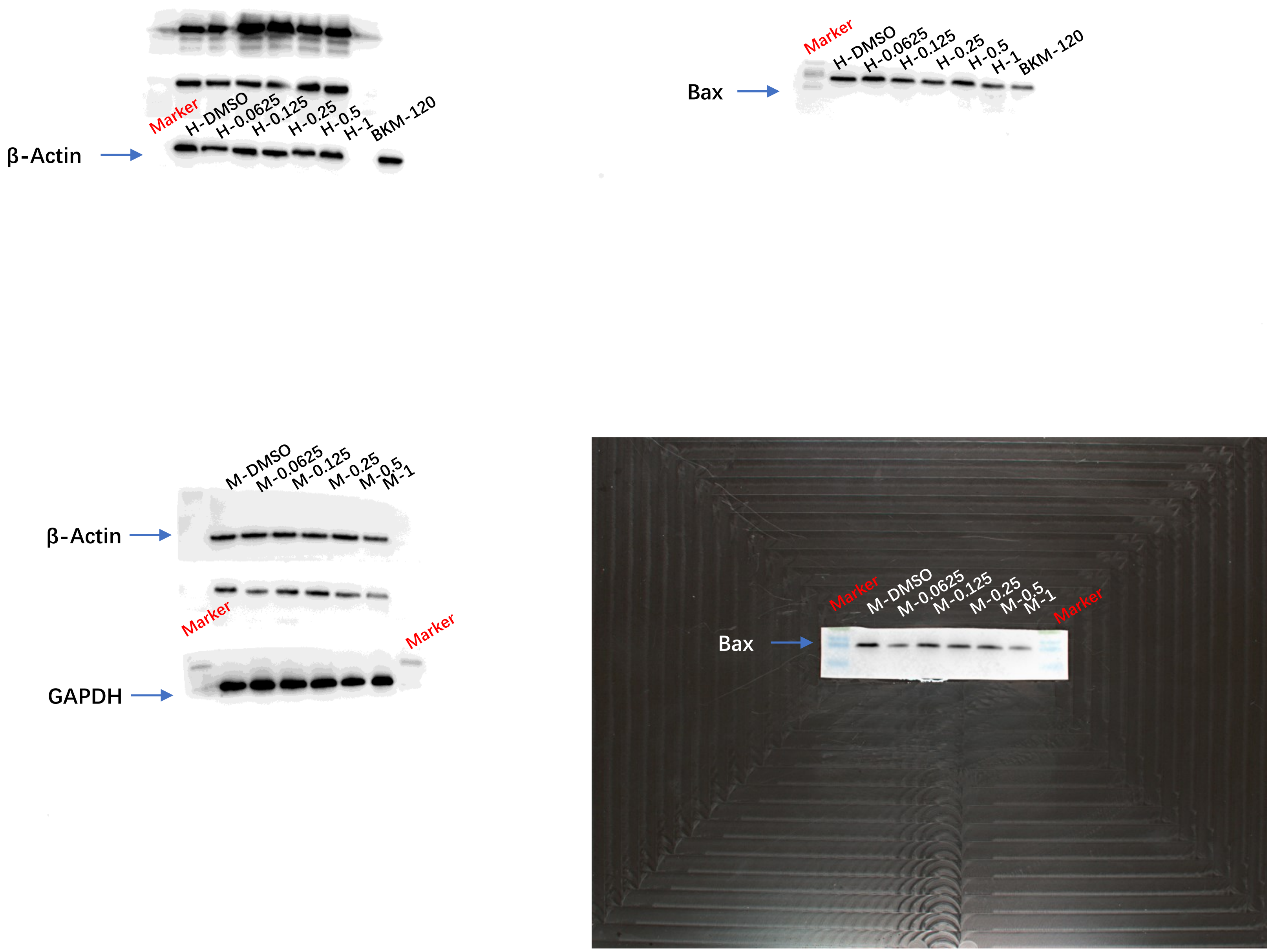
Marker
H-DMSO
H-0.0625
H-0.125
H-0.25
H-0.5
H-1
BKM-120
β-Actin
Marker
H-DMSO
H-0.0625
H-0.125
H-0.25
H-0.5
H-1
BKM-120
Bax
M-DMSO
M-0.0625
M-0.125
M-0.25
M-0.5
M-1
β-Actin
Marker
Marker
GAPDH
Marker
M-DMSO
M-0.0625
M-0.125
M-0.25
M-0.5
M-1
Marker
Bax

Figure. 6A

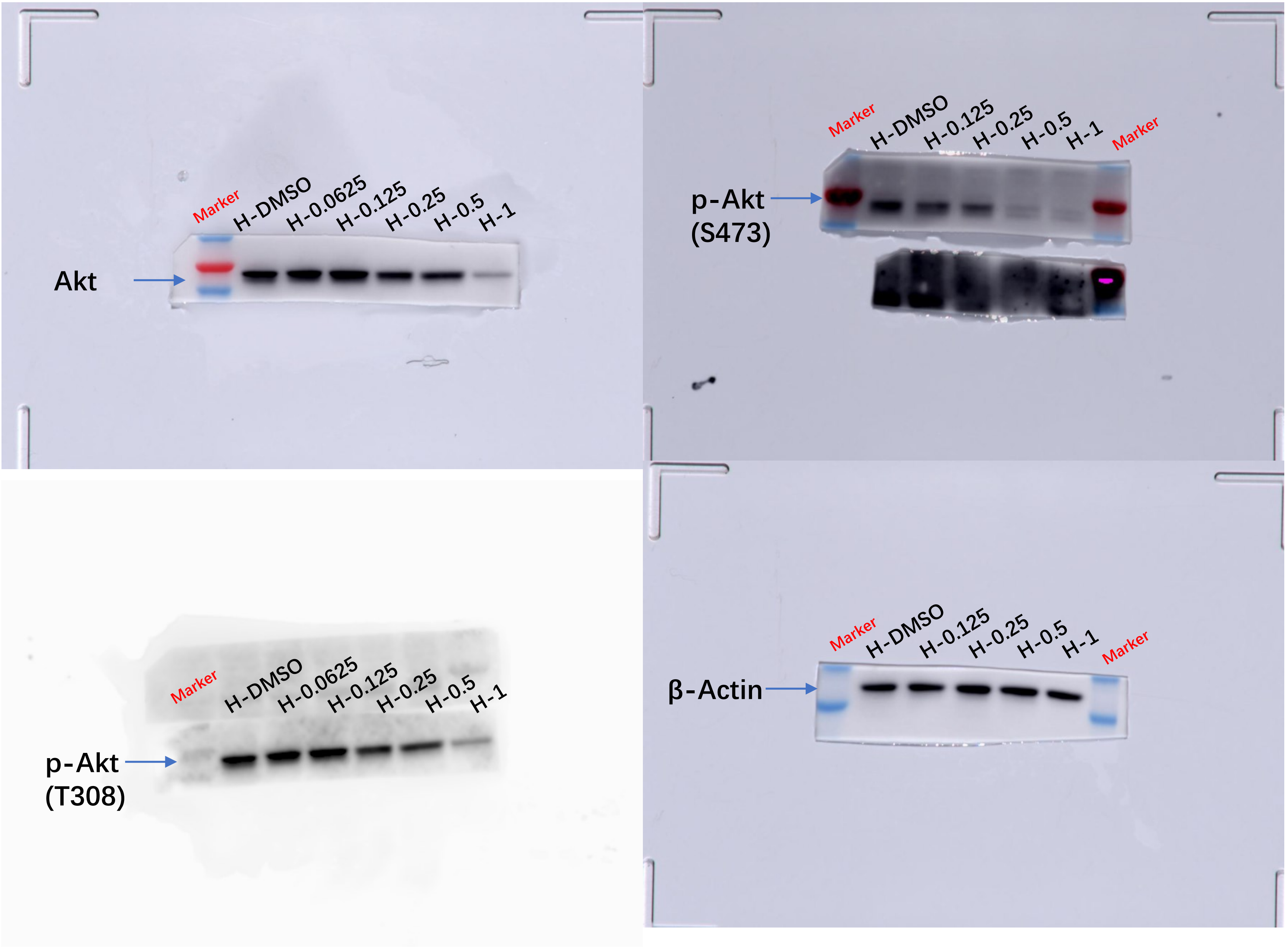
Marker
H-DMSO
H-0.0625
H-0.125
H-0.25
H-0.5
H-1
Akt
Marker
H-DMSO
H-0.125
H-0.25
H-0.5
H-1
Marker
p-Akt (S473)
Marker
H-DMSO
H-0.0625
H-0.125
H-0.25
H-0.5
H-1
p-Akt (T308)
Marker
H-DMSO
H-0.125
H-0.25
H-0.5
H-1
Marker
β-Actin

Figure. 6A

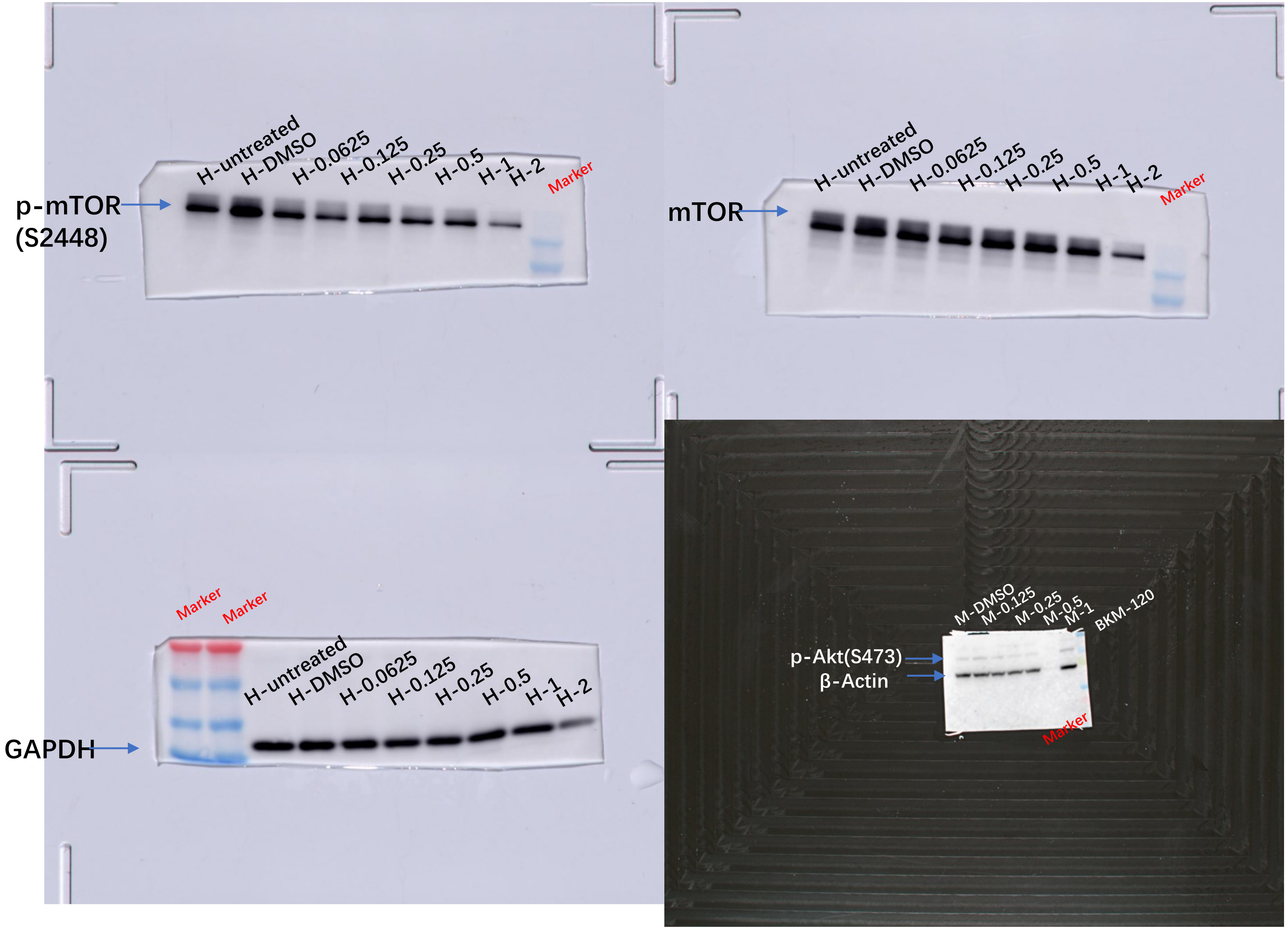
H-untreated
H-DMSO
H-0.0625
H-0.125
H-0.25
H-0.5
H-1
H-2
Marker
p-mTOR
(S2448)
mTOR
Marker
Marker
GAPDH
M-DMSO
M-0.125
M-0.25
M-0.5
M-1
BKM-120
p-Akt(S473)
β-Actin

Figure. 6A

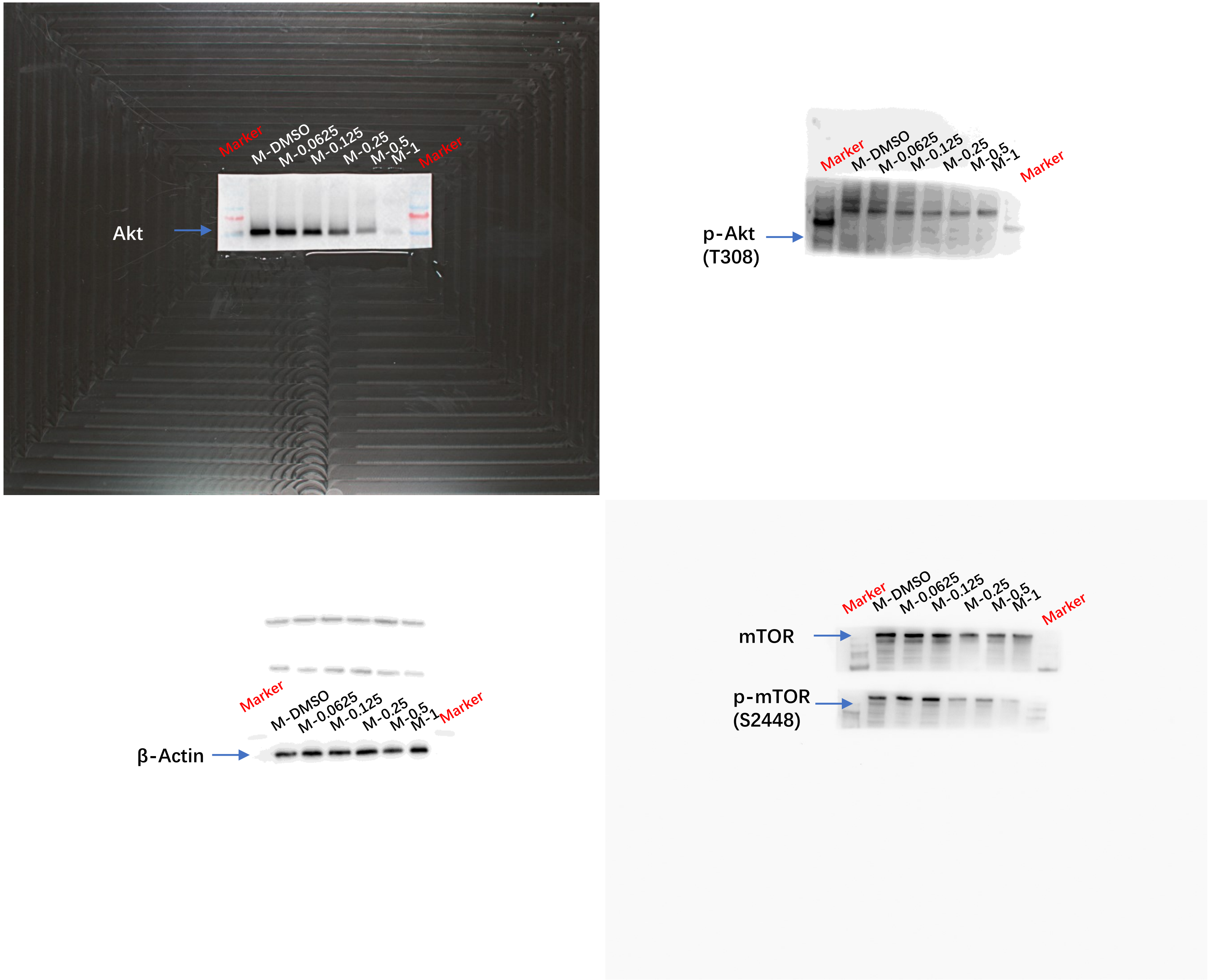
Marker
M-DMSO
M-0.0625
M-0.125
M-0.25
M-0.5
M-1
Marker
Akt
Marker
M-DMSO
M-0.0625
M-0.125
M-0.25
M-0.5
M-1
Marker
p-Akt
(T308)
Marker
M-DMSO
M-0.0625
M-0.125
M-0.25
M-0.5
M-1
Marker
β-Actin
Marker
M-DMSO
M-0.0625
M-0.125
M-0.25
M-0.5
M-1
Marker
mTOR
p-mTOR
(S2448)

Figure. 6B

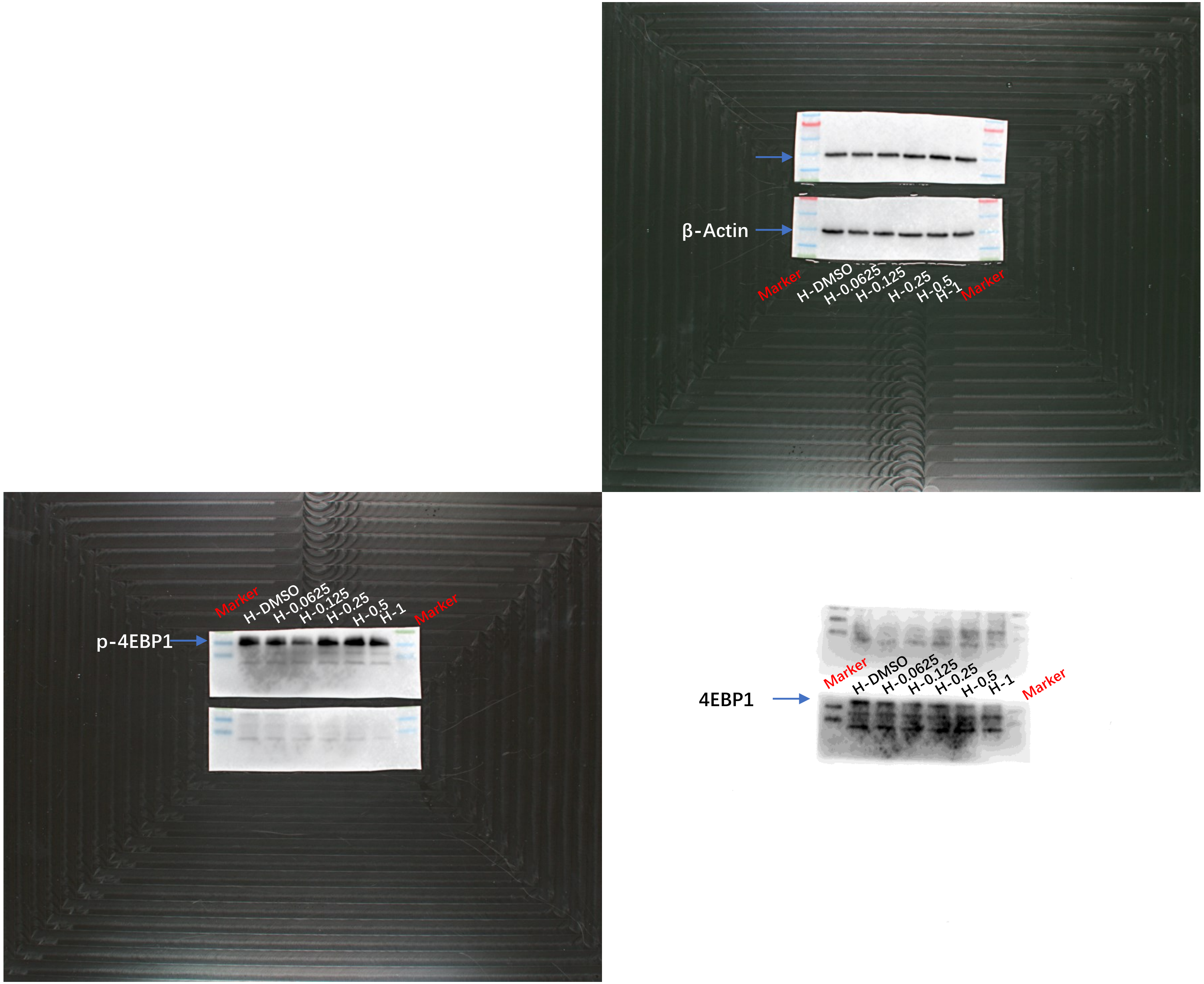
β-Actin
Marker
H-DMSO
H-0.0625
H-0.125
H-0.25
H-0.5
H-1
Marker
p-4EBP1
Marker
H-DMSO
H-0.0625
H-0.125
H-0.25
H-0.5
H-1
Marker
4EBP1
Marker
H-DMSO
H-0.0625
H-0.125
H-0.25
H-0.5
H-1
Marker

# Figure. 6B

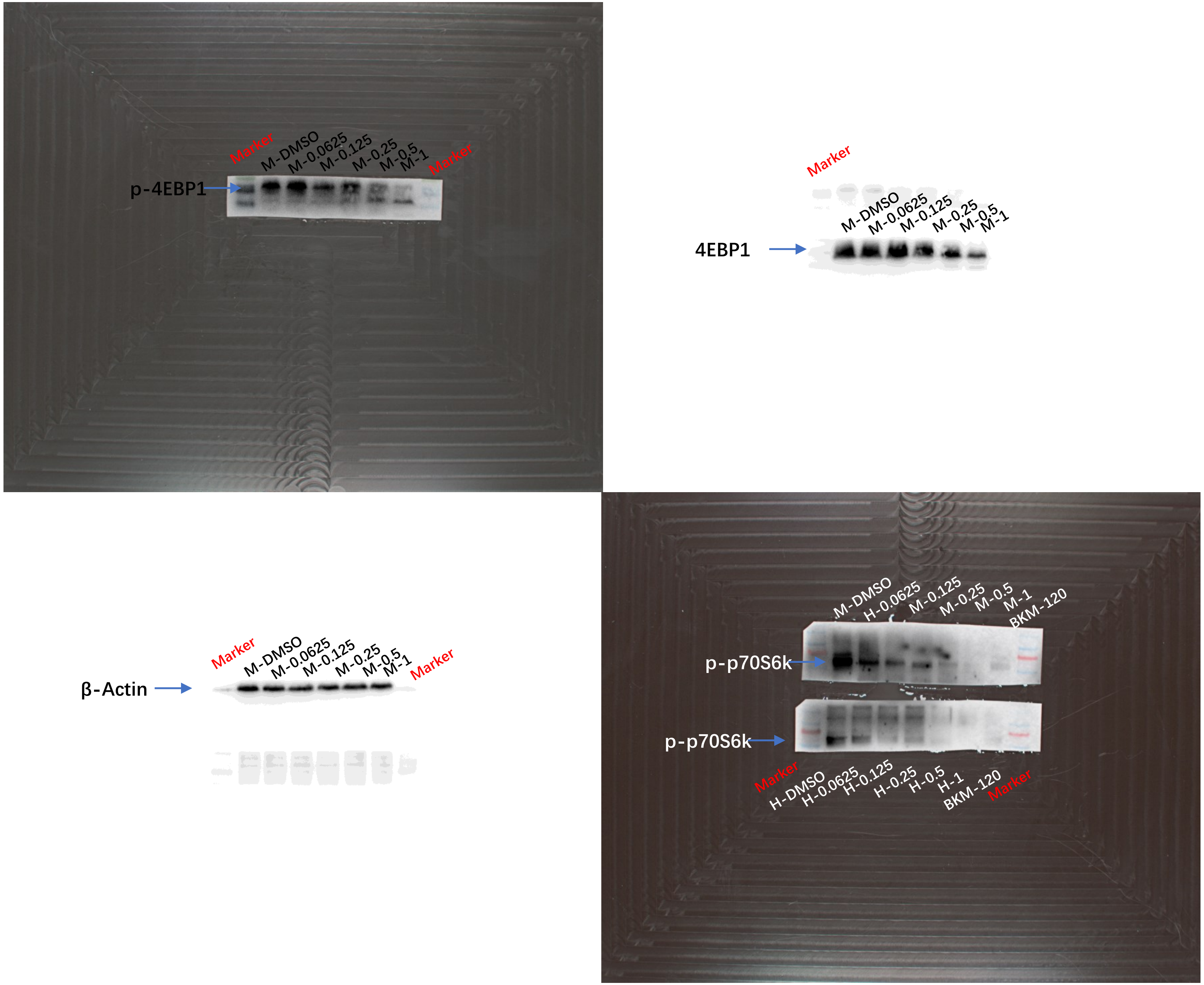

Marker
M-DMSO
M-0.0625
M-0.125
M-0.25
M-0.5
M-1
Marker
p-4EBP1
Marker
M-DMSO
M-0.0625
M-0.125
M-0.25
M-0.5
M-1
4EBP1
Marker
M-DMSO
M-0.0625
M-0.125
M-0.25
M-0.5
M-1
Marker
β-Actin
M-DMSO
H-0.0625
M-0.125
M-0.25
M-0.5
M-1
BKM-120
p-p70S6k
p-p70S6k
Marker
H-DMSO
H-0.0625
H-0.125
H-0.25
H-0.5
H-1
BKM-120
Marker
