# Supplementary material for: A pyridinesulfonamide derivative FD268 suppresses cell proliferation and induces apoptosis via inhibiting PI3K pathway in acute myeloid leukemia
Source: PLoS One. 2022 Nov 22;17(11):e0277893. doi: 10.1371/journal.pone.0277893 (PMC9681083; doi:10.1371/journal.pone.0277893)
Supplement: Attachment — Submitted filename: Response to Reviewers.docx [file pone.0277893.s013.docx]

Response to Reviewers

**Reviewer #1:**

Comment 1: in keywords: you do not need to include those that are already mentioned in the title (pyridinesulfonamide; acute myeloid leukemia; Apoptosis).

Response 1: Thank you for the suggestion about the key words. The key words that mentioned in the title has been replaced with the the following words: PI3K inhibitor, Haematological cancer and HL-60.

Comment 2: objectives are not well highlighted.

Response 2: Thank you for this valuable feedback. Our research have documented for the first time that a novel pyridinesulfonamide scaffold possesses significant anti-leukemic activity towards AML cells, the objective of our paper is about the early exploration of the mechanism of novel drug development. In the text of this paper, the objectives was not well highlighted due to the ways of language expression. We have further clarified this in the end of the Introduction section (lines 69-71), and the Conclusion section ( lines 457-461).

Comment 3: in some tests, in the text of materials and methods, it is not clear whether triplicates and 3 independent experiments were carried out, or only 3 independent experiments. They are only explained in the subtitles.

Response 3: Thank you for your questions. All the cells in each cell-based assay were seeded and harvested in triplicates, and at least three independent experiments were carried out in the CCK-8 cell proliferation assay and cell apoptosis assay. We have in added examinations in the text of materials and methods (lines 104-105, lines 120-121 and lines 145-146).

Comment 4: in figure 4c the name of the cell lines was missing.

Response 4: Thanks for pointing out about the missing details, the name of cell lines has been added in Figure 4C.

Comment 5: the work has been done with two cell types, but in RNA-seq only one cell line was chosen, but there was no explanation for this.

Response 5: We appreciate for this question. Based on the previous studies of compound FD268, we found that FD268 has potential AML cell proliferation inhibitory activity, and further screening assay showed that the HL-60 cell line was more sensitive to compound FD268, so in the subsequent mechanistic explorations we chose HL-60 for the RNA-seq analysis.

**Reviewer #2:**

Comment 1: Line 90: repeating words “and and”.

Comment 2: Line 108: Should read “…and treated with FD268…”

Comment 3: Line 178: Should read “…plates and treated with FD268 (0.125, 0.5 µM) or DMSO for 24 h. Cells were harvested…”

Comment 4: Line 225: Should read “The values were shown as the…”

Response 1-4: Thanks for the kind comments. The tense and repetition mistakes have been corrected in the manuscript.

Comment 5: Lines 249-250: Fig. 3C: there is no dose dependent inhibition of cleavage of caspase 3 and PARP, but increase instead. The sentence should be corrected as “…proteins were consequently examined by western blot assay, which showed a dose dependent increase of both caspase-3 and PARP cleavage (Fig 3C).”

Response 5: Thanks for the comments. In Figure. 3C, the western blot was applied to detect cleaved PARP, cleaved caspase-3 and caspase-3 as indicators of apoptotic cell death. The cleavage of caspase-3 and PARP increased dose dependently. We have changed the sentences as suggested. (Lines 249-250)

Comment 6: Line 258: Should read “Statistical significance is represented with asterisks *P < 0.05 and ***P < 0.001.”, as there is no **P < 0.01. It might be also better to use white background for S phase data, as labels/asterisks are barely visible in Figure 2B.

Response 6: Thanks for the valuable suggestion and we have removed the “**P < 0.01” in statistical significance annotation of Figure 2. The background for S phase data bar have been re-edited to highlight labels/asterisks.

Comment 7: Figure 4C: Indicate which blots represent HL-60 and MOLM-16 cells. Label for cell lines is missing.

Response 7: Thanks for pointing out about the missing, the name of cell lines has been added in Figure 4C.

Comment 8: Line 288: The authors should mention here that the protein levels of other markers of apoptosis, e.g. Bcl-2, Bax and Bcl-XL, did not change significantly in FD268-treated cells.

Response 8: Thanks for this kind suggestion. At protein level, FD268 increased the expression of Bad, meanwhile inhibited Mcl-1 in both HL-60 and MOLM-16 dose-dependently. While, the protein levels of other markers of apoptosis, Bcl-2, Bax and Bcl-XL, did not change significantly in FD268-treated cells. We have added a sentence to to clarify this. (Lines 291-292)

Comment 9: Figure 5C: Label of MOLM-16 has to be corrected (swapped letters). The same applies to Figure 6A and 6B.

Response 9: Thanks for pointing out the mistakes, the label of MOLM-16 have been corrected in Figure 5C, 6A and 6B.

Comment 10: Figure 9: Shouldn´t the cleaved caspase-3 and PARP lead to cell death/apoptosis, instead of cell survival (check the bottom right corner)?

Response 10: Thank you for this valuable comment. In this diagram, we meant to show that the compound FD268 induced apoptosis through the activation of caspase-3 and PARP cleavage, so the label “cell Survival” have been changed into “Cell Apoptosis”.
